# Combining DNA Barcoding and Chemical fingerprints to authenticate Lavender raw material

**DOI:** 10.1111/ics.12757

**Published:** 2021-12-23

**Authors:** Florian Philippe, Nelly Dubrulle, Benjamin Marteaux, Brice Bonnet, Patrick Choisy, Jean‐Yves Berthon, Laurence Garnier, Nadine Leconte, Sandrine Milesi, Pierre‐Yves Morvan, Alex Saunois, Jian‐Sheng Sun, Sandrine Weber, Nicole Giraud

**Affiliations:** ^1^ DNA Gensee 17 rue du lac saint andré Le Bourget du Lac 73370 France; ^2^ L’Oréal Aulnay‐sous‐Bois France; ^3^ LVMH Recherche Saint Jean Le Braye France; ^4^ Greentech Saint‐Beauzire France; ^5^ Nuxe Boulogne Billancourt France; ^6^ Laboratoire Clarins Pontoise France; ^7^ Codif Saint‐Malo Cedex France; ^8^ Sederma Le Perray‐en‐Yvelines France; ^9^ Structure et Instabilite des Génomes Muséum national d’Histoire naturelle CNRS INSERM 43 rue Cuvier Paris 75005 France

**Keywords:** barcoding, bioinformatics, chemical analysis, lavender, plant authentication

## Abstract

**Objective:**

This study was initiated and conducted by several laboratories, 3 of the main cosmetic ingredient suppliers and 4 brands of cosmetics in France. Its objective is to show the interest and robustness of coupling chemical and genetic analyses in the identification of plant species. In this study, the *Lavandula* genus was used.

**Methods:**

In this study, we used two analytical methods. Chemical analysis from UHPLC (ultra‐high‐performance liquid chromatography) and genetic analysis from barcoding with genetic markers.

**Results:**

Eleven lavender species were selected (botanically authenticated) and analysed. The results show that three chemical compounds (coumaric acid hexoside, ferulic acid hexoside and rosmarinic acid) and three genetic markers (*RbcL*, *trnH*‐*psbA* and *ITS*) are of interest for the differentiation of species of the genus *lavandula*.

**Conclusion:**

The results show that the combination of complementary analytical methods is a relevant system to prove the botanical identification of lavender species. This first study, carried out on a plant of interest for cosmetics, demonstrates the need for authentication using a tool combining genetic and chemical analysis as an advance over traditional investigation methods used alone, in terms of identification and authentication reliability.

## Résumé

**Objectif:**

Cette étude a été lancée et menée par plusieurs laboratoires, trois des principaux fournisseurs d’ingrédients cosmétiques et quatre marques de cosmétiques en France. Son objectif est de montrer qu’associer les analyses chimiques et génétiques dans l’identification des espèces végétales présente un intérêt et est une approche solide. Dans cette étude, c’est le *genre* Lavandula qui a été utilisé.

**Méthodes:**

Dans cette étude, nous avons fait appel à deux méthodes analytiques. L’analyse chimique, à partir de la chromatographie en phase liquide à haute performance (ultra‐high‐performance liquid chromatography, UHPLC), et l’analyse génétique en procédant à un codage à barres avec des marqueurs génétiques.

**Résultats:**

Onze espèces de lavande ont été sélectionnées (authentifiées du point de vue botanique) et analysées. Les résultats montrent que trois composés chimiques (acide coumarique hexoside, acide ferulique hexoside et acide rosmarinique) et trois marqueurs génétiques (*RbcL*, *trnH*‐*psbA* et *ITS*) présentent un intérêt pour la différenciation des espèces du genre *lavandula*.

**Conclusion:**

Les résultats montrent que la combinaison de méthodes analytiques complémentaires est un système pertinent pour prouver l’identification végétale des espèces de lavande. Cette première étude, réalisée sur une plante qui offre un intérêt pour les cosmétiques, démontre la nécessité de procéder à une authentification à l’aide d’un outil qui conjugue analyse génétique et chimique ; elle représente une avancée par rapport aux méthodes d’investigation traditionnelles utilisées seules, en termes d’identification et de fiabilité de l’authentification.

## INTRODUCTION

Nowadays, the sourcing of plant raw materials is a major issue for the cosmetic and nutraceutical industries for different reasons: security, marketing and ethics [1, 2]. It is also necessary to evaluate the plant composition to detect other contaminating plant species. The control by chemical analysis to detect potentially harmful compounds resulting from harvest conditions (phytosanitary products or plant pathogen contents) or other chemical molecules used to decrease the cost production is also important [3]. All plant species are susceptible to be adulterated. For example, the substitution of Japanese star anise (*Illicium anisatum*) can be done with Chinese star anise (*Illicium verum*) [4]. This confusion in health drinks can cause neurological and gastrointestinal symptoms for infants [5]. Many cases of fraud have been detected in lavender essential oil and honey [6, 7].

*Lavandula* (L.) species are endemic of different world areas/northern Africa, the Mediterranean, south‐west Asia, Arabia and western Iran [8, 9]. *Lavandula* genus (Lamiaceae) includes 39 species and a multitude of cultivars and field varieties exceeding 400 [10, 11, 12]. The majority of the species in this genus are constituted by small evergreen shrubs, with aromatic foliage and flowers [13]. Due to these particularities, the majority of lavenders are the subject of several scientific studies. This plant has great economic importance in perfumery and cosmetics, food manufacturing and aromatherapy [14, 15]. To increase the level of specific chemical molecules in plants, new cultivars have emerged through classical breeding programmes, for instance, fertile *Lavandula* × intermedia cultivars have been generated by crossbreeding of *L. angustifolia* and *L. latifolia* [9, 16]. Consequently, the presence of many cultivars requires to increase controls. To distinguish them, three techniques have been developed, previously the chemical identification with HPLC‐UV‐MS (high‐performance liquid chromatography with mass spectrometry) or GC‐MS (gas chromatography–mass spectrometry) and more recently genetic identification with barcoding [17, 18, 19].

European Medicines Agency (EMA) suggests that specific identification tests for substitute and adulterant detection must be performed. One can either use a combination of separate chromatographic approaches (HPLC with TLC‐densitometry) or combine different approaches into a single procedure (HPLC‐UV, HPLC‐MS or GC‐MS) (European Medicines Agency, 2006). Concerning the lavender essential oil, it is mainly obtained from *L. angustifolia*. This species is the most expensive lavender because the quality of its essential oil is better compared to other *Lavandula* species [20, 21]. It can be falsified with other oils (lavandin or spike) or with synthetic molecules such as linalyl acetate [22]. To authenticate the lavender oil, the relative abundance of compounds such as linalool, linalyl acetate, borneol, camphor and 1,8‐cineole is quantified by GC‐MS analysis [23]. However, the quality of the essential oil and its chemical composition are depending on the geographic region of origin [24]. Moreover, their chemical authentication and molecule distribution can be phenotypically influenced by the environmental conditions which might lead to a problem in botanical origin identification [25, 26].

More recently, a new method involving genetic analysis has emerged, barcoding analysis [27, 28, 29, 30]. This method is an approach for species identification using a specific part of nuclear, mitochondrial or chloroplastic DNA sequences, to identify an organism [27, 31]. Currently, DNA barcoding is considered as an efficient technique used to identify cases of adulteration and to specifically identify the plant species present in a raw material mixture, plant or food product [30, 32, 33]. In 2009, the The Consortium for the Barcode of Life (CBOL) proposed to identify a specific universal genetic region for the identification of plant species (e.g. in CBOL Plant Working Group et al., 2009). However, this project has been hindered by the lack of genetic data and the presence of many cultivars and hybrids. Currently to identify plant species by barcoding analysis, several genes are used, plastid‐encoded large subunit of RuBisCO (rbcL), maturase‐K (matK) or plastid intergenic spacer trnH‐psbA and the nuclear internal transcribed spacer (ITS) [34, 35]. Concerning the *Lavandula* genus, the presence of diverse lavender species implicates the need for a reliable botanical identification system. For lavender, many barcoding analyses used a single marker to build the *Lavandula* genus phylogenetic relationships’ tree [36, 37]. Recently, a study has used another type of genetic markers, the single sequences repeat markers (SSR) from ESTs (Expressed Sequence Tag) of *L. angustifolia* and L. × intermedia to discriminate this species [38]. But genetic markers seem to be not sufficient in differentiation of closely related species because there are many conserved regions of the transcribed sequences.

To control the quality of lavender raw materials, it is necessary to confirm their identity to ensure the species distinction and traceability. In this study, we proposed to combine chemical analysis (UHPLC) and genetic analysis (barcoding) to identify different lavender species. This study is based on botanically authenticated samples from botanical gardens.

## MATERIALS AND METHODS

### Plant materials

Ten samples of *Lavandula* (L.): *L*. *angustifolia* (varieties White and Blue), *L*. *angustifolia* × *L*. *dentata* (*L*. *allardi*), *L*. *canariensis*, *L*. *dentata*, *L*. × *intermedia* (varieties *‘Abrial’* and *‘Grosso’*), *L*. *latifolia*, *L*. *pinnata*, *L*. *stoechas* and one outgroup species *Perovskia atriplicifolia*—were collected from two French botanical gardens, the “Musée de la lavande Ardèche” and the “Musée de la Parfumerie of Grasse.” All samples were dried and stored in silica gel at room temperature (22–25°C). Botanical authentication vouchers were established by the company Botanicert, Grasse.

### DNA barcoding

#### DNA extraction, amplification and sequencing

Genomic DNA was extracted from dried leaves and stem samples (~100 mg) using NucleoMag Plant kit^®^ (Macherey nagel^®^) according to the manufacturer's protocol. The quantity and quality of DNA extracts were quantified and verified by spectroscopy using a SimpliNano^®^ microvolume spectrophotometer (GEe HealthCare^®^). Two barcodes regions were chosen in the database “BOLD Systems v3” and one barcode primer was designed using primer3plus software (Table 1) [39, 40]. *rbcl* and *trnH*‐*psbA* primers have ambiguous nucleotide bases because they allow the identification of a large number of plant species. All three barcodes were amplified by PCR (polymerase chain reaction). The PCR conditions were 95°C for 10 min, followed by 35 cycles at 95°C for 30 s, primer melting temperature depending on the primers for 30 s and 72°C for 1 min, with a final incubation at 72°C for 7 min. The amplified PCR products were controlled using a QIAxcel system (Qiagen^®^) to be sure that the DNA has been amplified and is in a sufficient quantity for sequencing. Amplified DNA products were directly sequenced with standard Sanger sequencing protocols on Applied Biosystems SeqStudio Genetic Analyzer^®^ (Thermo Fisher Scientific^®^).

**TABLE 1:** Markers and their characteristics

Marker name	Genetic region	Primer name	Sequence (5′−3′)
B1	*rbcl*	rbcLbF	AGACCTWTTTGAAGAAGGTTCWGT
		rbcLbR	TCGGTYAGAGCRGGCATRTGCCA
B2	*trnH‐psbA*	psbA3′ f	GTTATGCATGAACGTAATGCTC
		trnHf_05	CGCGCATGGTGGATTCACAATCC
Lav1	*ITS*	LavF1	CTGCGGAAGGATCATTGT
		LavR1	TTGATATGCTTAAACTCAGC

#### Genetic data analysis

Geneious^®^ software was used to assemble raw data, create a consensus sequence with a combination of F and R sequences [41]. All of the sample sequences were selected to construct a tree with UPGMA method. Bootstrap tests were conducted using 1000 replicates to estimate the identification efficacy of phylogenetic relationships.

### UHPLC analysis

#### Extraction solvent choice and sample preparation

A dried powdered sample test was precisely weighed (1 g) and introduced in 10 ml of different solvents: H_2_O, EtOH/H2O (3:7 w/w), EtOH/H2O (3:1 w/w), MeOH and DMSO (DiMethyl SulfOxide). Extractions were carried out using an ultrasonic extraction at room temperature during 10 min. To extract the majority of metabolites, the solution was filtered through a cellulose membrane (0.22 µm). EtOH (3:1) was selected as extraction solvent because it provides the highest level of compounds.

#### Instrumentation and analytical Conditions

A specific method was developed to provide a good separation of the major part of the non‐targeted compounds. UHPLC‐DAD analysis was performed with 1 μl injection in Kinetex C18 column (Phenomenex 2.6 μm, 150 × 2.1 mm). The following mobile phase was used with a flow rate of 0.6 ml/min: start with 5% of acetonitrile with 0.01% formic acid (B) to 40% for 9 min then 40% to 100% of (B) from 9 to 15 min and 100% during 5 min, where (A) is 0.01% formic acid in water and (B) is acetonitrile with 0.01% formic acid. The UV detection was performed by Waters ACQUITY^®^ DAD and the absorbance was measured at “max plot” (200–450 nm). Mass detection was performed by Waters ACQUITY^®^ SQD1 Electro Spray Ionization in positive mode, with a source temperature of 150°C, a desolvation temperature of 500°C and a capillary voltage of 3.5 kV. Two voltage cones were used simultaneously (10 and 40 V). For peak identification, different analytical standards were used through comparison with retention time and mass spectra.

## RESULTS

### UHPLC analysis

#### Chemical marker identification and selection

**FIGURE 1:**
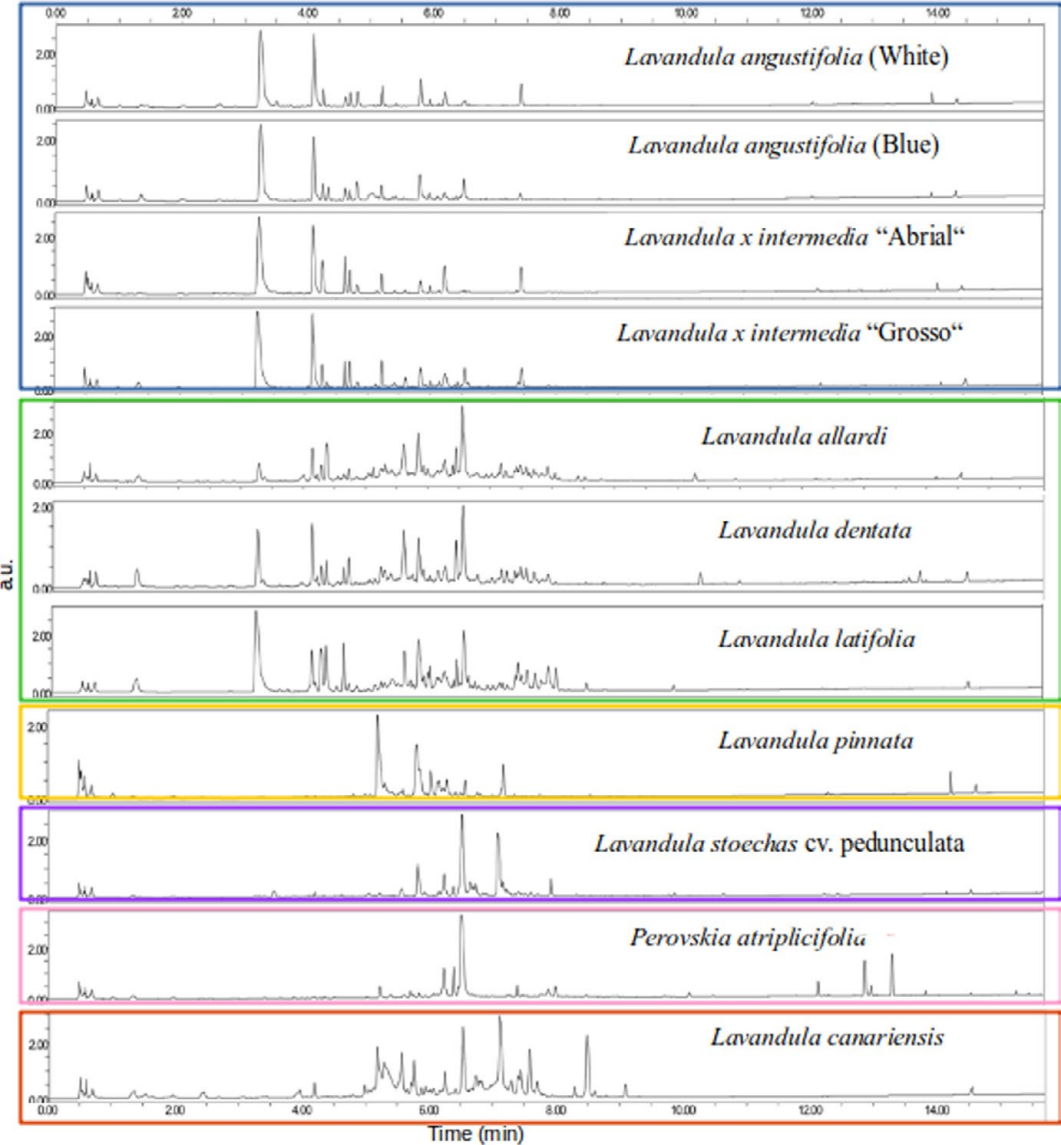
UHPLC‐DAD chromatograms of 11 Lavander samples. The blue, green, yellow, purple, pink and red rectangles correspond to the representative molecules of cluster 1, 2, 3, 4 and 5 respectively

The ethanol extract constituents of the lavender samples were determined by UHPLC‐UV‐MS. EtOH (3:1) was selected as extraction solvent because it provides the highest level of non‐target compounds. Chromatogram analysis detected 99 major compounds present in the samples (Data S1). Comparison of chromatograms of the different samples (triplicate analysis for each compound) allowed to identify 7 compounds with significant peak area values in at least one of lavender analysed samples (Figure 1). Four compounds belonged to the hydroxycinnamic acid family, compound n°8 (coumaric acid hexoside), compound n°14 (ferulic acid hexoside), compound n°21 (a molecule derived from coumaric acid) and compound n°53 (rosmarinic acid). Two others compounds belonged to the family of phenylpropanes, compound n°15 (glucoside of hydroxycinnamic acid) and compound n°16 (derivative from cinnamic acid). Finally, the last compound was part of the flavone family, compound n°37 (luteoline‐7‐O‐glucuronide).

#### Chemical profile

Figure 1 shows the chromatography profiles of the samples. The set of molecules identified is presented in Data S1. Among the 99 compounds measured by the analyses of the 11 samples, metabolites, which have the highest molecular weight (Data S1), were identified.

Different and similar profiles are shown in Figure 1. The samples of *Lavandula angustifolia* (cv. “White” and cv. “Blue”) and *Lavandula* × *intermedia* (cv. “Abrial” and cv. “Grosso”) have very similar chemical profiles. These samples showed two main peaks at retention times of 3.23 min and 4.10 min (blue rectangle). Samples of *Lavandula allardi*, *Lavandula dentata* and *Lavandula latifolia* showed quite similar chemical profiles. They had the particularity of presenting important peaks at retention times of 4.31 and 6.52 min (green rectangle). The chemical profiles of the samples of *Lavandula pinnata* (orange rectangle), *Lavandula stoechas* (purple rectangle), *Perovskia atriplicifolia* (pink rectangle) and *Lavandula canariensis* (red rectangle) appeared to be unique.

#### Cluster identification

The two important peaks for samples of *Lavandula angustifolia* (cv. “White” and cv. “Blue”) and *Lavandula* × *intermedia* (cv. “Abrial” and cv. “Grosso”) corresponded to coumaric acid hexoside (compound 8, retention time 3.23 min) and ferulic acid hexoside (compound 14, retention time 4.10 min). The presence of these molecules was the first selection criterion. These samples constituted cluster 1 (Table 2). In this cluster, species *Lavandula angustifolia* cv. *“Blue”* and *Lavandula* × *intermedia* cv. *‘*‘Grosso’’ presented very similar profiles. They both presented a high proportion of rosmarinic acid (compound 53, retention time 6.52 min) (criterion 2), two molecules derived from cinnamic acid (hydroxycinnamic acid glucoside (retention time 4.31 min, compound 15) and one unknown substance which is not present in the database (retention time 4.40 min, compound 16)) (criterion 3) compared to the other two species in this cluster.

**TABLE 2:** Criteria for differentiating samples

Samples	Cluster	Criteria						
		1	2	3	4	5	6	7
*Lavandula angustifolia* cv. “White”	1	x					x	
*Lavandula angustifolia* cv. “Blue”		x	x	x			x	
*Lavandula* × *intermedia* cv. “Abrial”		x					x	
*Lavandula* × *intermedia* cv. “Grosso”		x	x	x			x	
*Lavandula allardi*	2		x	x				
*Lavandula dentata*		x	x	x				
*Lavandula latifolia*			x	x				
*Lavandula pinnata*	3		x		x			
*Lavandula canariensis*	4		x		x	x		
*Lavandula stoechas* cv. “Pedunculata”	5		x		x	x	x	
*Perovskia atriplicifolia*	6		x		x	x		x

Note

Each criterion was selected by major presence or absence of compounds. The “x” validates the criterion. The samples were classified into each cluster according to the following criteria. Criteria 1: compounds 8 (Coumaric acid hexoside) and 14 (Ferulic acid hexoside) major presence, Criteria 2: Compound 53 (rosmarinic acid) major presence, Criteria 3: presence of compounds 15 (hydroxy hydrocinnamic acid glucoside) and 16 (cinnamic acid derivative), Criteria 4: absence of compounds 15 and 16, Criteria 5: presence of depsides family, Criteria 6: presence of compound 93 (Linalyl acetate), Criteria 7: presence of diterpenes family.

The species *Lavandula allardi*, *dentata* and *latifolia* also showed similar chemical profiles. They were the cluster 2 (Table 1). They presented criterion 2, that is a high proportion of rosmarinic acid, but also criterion 3, which is the presence of two molecules derived from cinnamic acid. In this cluster, the species *Lavandula dentata* also presented coumaric acid hexoside (compound 8, retention time 3.23 min) and ferulic acid hexoside unlike the other two species.

The species *Lavandula pinnata*, *Lavandula stoechas* cv. “Pedunculata,” *canariensis* and *Perovskia atriplicifolia* presented no traces of the two molecules derived from cinnamic acid (criterion 4). This criterion was characteristic of cluster 3 (Table 1). The species *Lavandula stoechas* cv. Pedunculata, *canariensis* and *Perovskia atriplicifolia* were the only species which show depsides (compound 51 (6.45 min), 55 (6.66 min), 56 (6.71 min), 57 (7.08 min), 59 (7.16 min), 66 (7.56 min), 73 (8.27 min), 74 (8.46 min) and 77 (9.07 min)), characteristic of cluster 4. The species *Lavandula stoechas* cv. “Pedunculata” presented linalyl acetate (12.79 min, compound 93). This criterion differentiates it from the other samples and classifies it in cluster 5. *Perovskia atriplicifolia* is the only species studied which presents diterpenes 94 (12.85 min), 95 (12.85 min) and 96 (13.28 min)) characteristic molecules of cluster 6.

### DNA barcoding analysis

#### Bold DNA barcoding markers results

**FIGURE 2:**
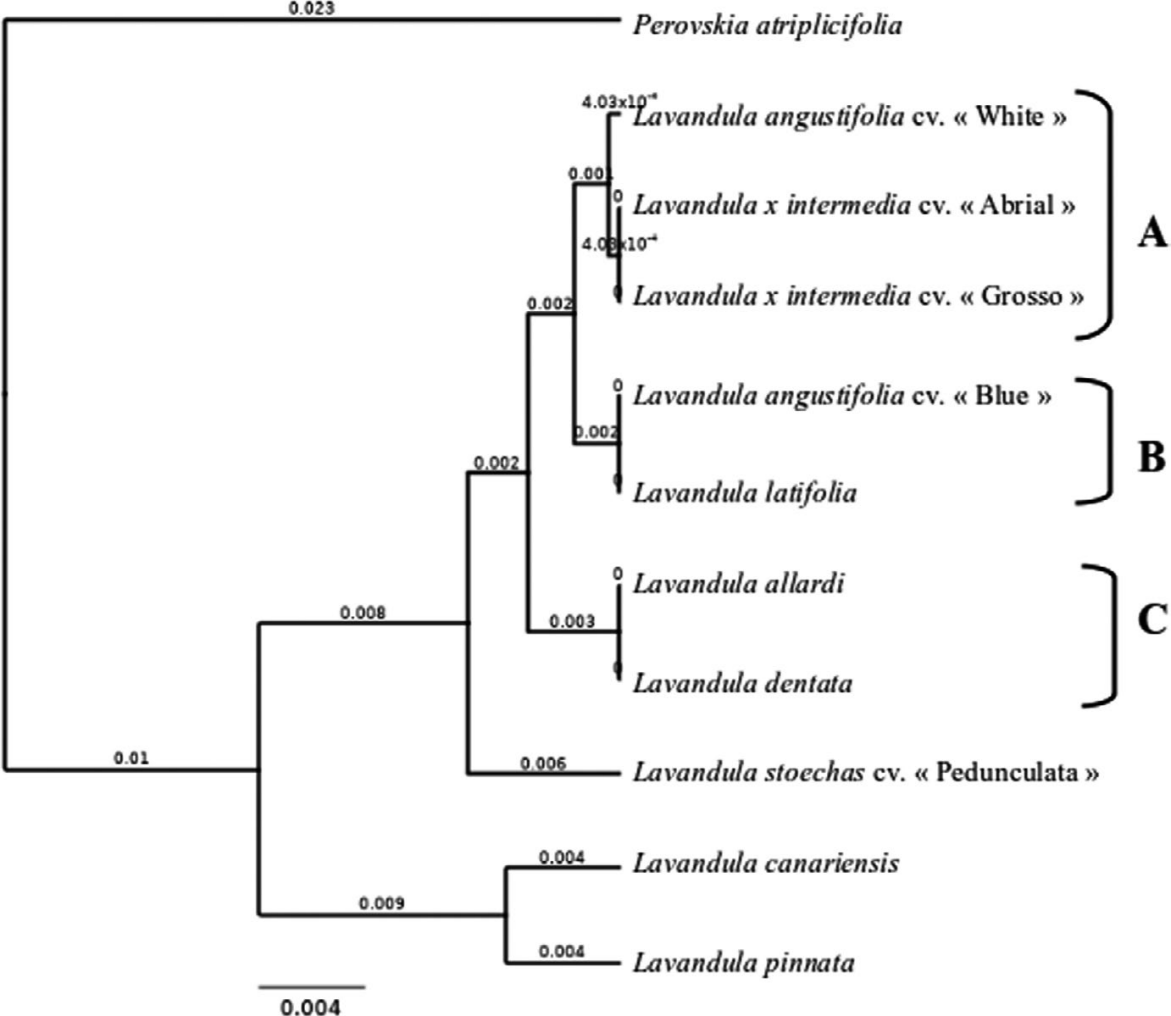
Phylogenetic tree of plant samples with rbcl and *trnH*‐*psbA* genetic markers. Phylogenetic tree built with chimeric sequences of rbcl and *trnH*‐*psbA* genetic markers results using Geneious^®^ software with UPGMA method. Branch support was based on 1000 bootstrap replicates and is shown at the nodes. The bar represents 0.004 substitutions per site

From the literature, two potential genetic markers (*rbcl* (*RuBisCo* large subunit) (B1) and *trnH*‐*psbA* (B2)) were selected in the BOLD Database (Barcode of Life Data System). All samples were successfully amplified from total DNA and sequenced. Sequence analysis showed the same length between samples for each marker, respectively, *rbcl* had 690 bp and *trnH*‐*psbA* had 273 bp. To analyse the result, an alignment was performed to identify the discriminated species. Moreover, a phylogenetic tree was constructed based on the compilation of *rbcl* and *Trnh*‐*psba* (Figure 2). The UPGMA tree, which was carried out with the *rbcl* and *trnH*‐*psbA* markers, showed that 4 species can be fully differentiated, *Lavandula pinnata*, *Lavandula stoechas* cv. “Pedunculata,” *Perovskia atriplicifolia* and *Lavandula canariensis*.

This tree also revealed three differentiated clusters (A, B and C). These clusters gather samples that have the same genetic sequences for each genetic marker studied. The first cluster, A, included *Lavandula angustifolia* (white variety) and two lavandin (*Lavandula x intermedia* cv. “Abrial” and *Lavandula* × *intermedia* cv. “Grosso”). The second cluster, B, was composed of *Lavandula angustifolia* cv. “Blue” and *Lavandula latifolia*. The third cluster, C, included *Lavandula allardi* and *Lavandula dentata*.

#### Specific Lavander DNA barcoding marker results

**FIGURE 3:**
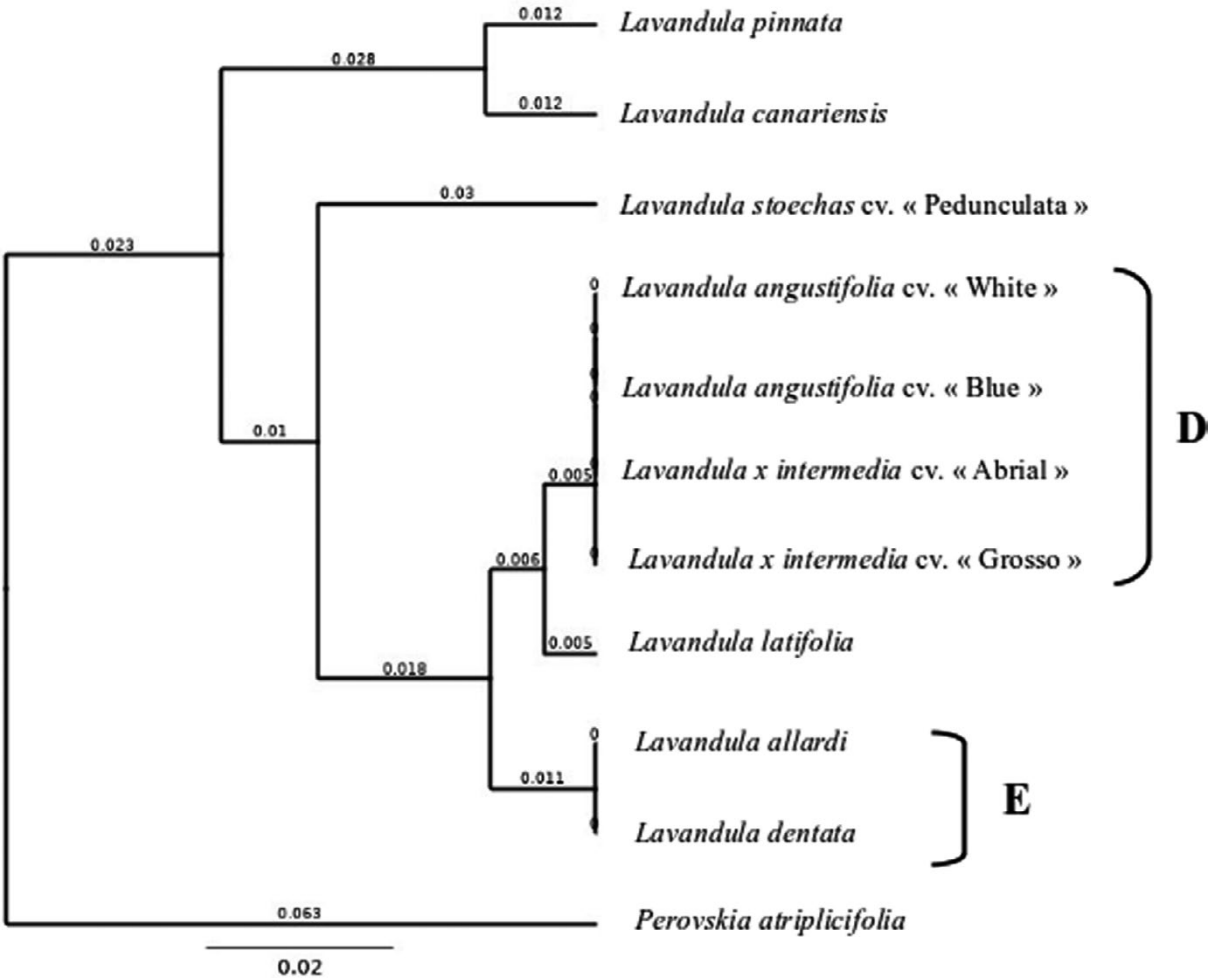
Phylogenetic tree of plant samples with Lav genetic marker. Phylogenetic tree built with compilation of rbcl and *trnH*‐*psbA* markers genetic data using Geneious^®^ software with UPGMA method. Branch support was based on 1000 bootstrap replicates and is shown at the nodes. The bar represents 0.004 substitutions per site

To identify more lavender species, a specific marker for the genus *Lavandula* was created. This marker was located in the *ITS* (Internal transcribed spacer) locus (Lav marker). This marker was created thanks to GenBank^®^ database chloroplast sequences of genus *Lavandula*. All 11 samples were successfully amplified and sequenced with this marker. The *Lav* marker presented a size of 490 bp. To identify the species differentiated with this marker, a UPGMA phylogenetic tree was created (Figure 3). This tree was built using chimeric genetic sequences. It showed that 5 species can be fully differentiated, *Lavandula pinnata*, *Lavandula stoechas* cv. “Pedunculata,” *Perovskia atriplicifolia*, *Lavandula canariensis* and *Lavandula latifolia*. As in Figure 2, two new clusters, D and E, could be identified. They gather samples that have the same genetic sequences for the ITS marker. Clusters D includes *Lavandula angustifolia* cv. “White,” *Lavandula angustifolia* cv. “Blue” and the two lavandins (*Lavandula* × *intermedia* cv. "Abrial" and *Lavandula* × *intermedia* cv. "Grosso"). Cluster E was composed of *Lavandula allardi* and *Lavandula dentata*.

**FIGURE 4:**
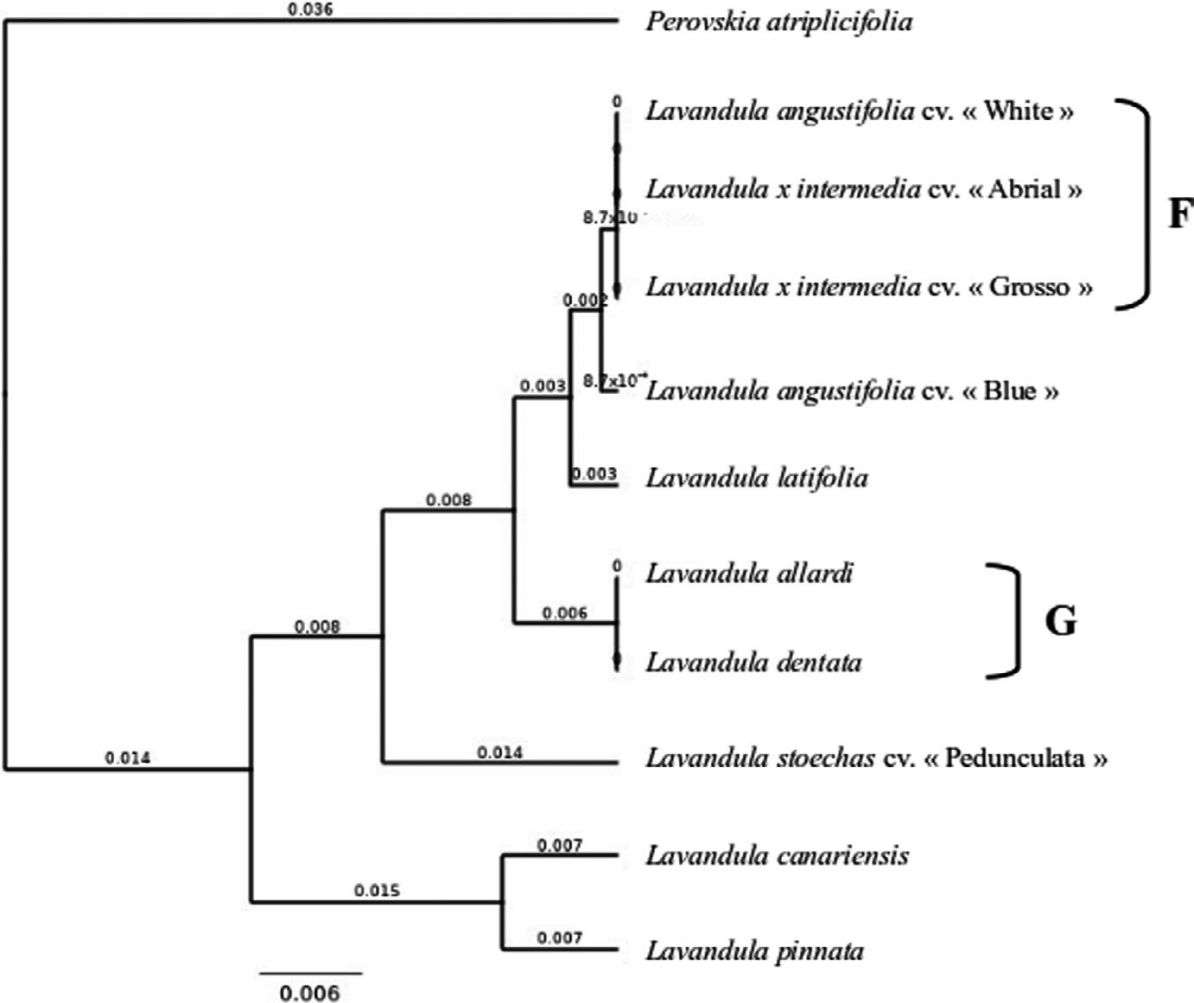
Phylogenetic tree of plant samples with a chimeric construction of all genetic marker results. Phylogenetic tree built with all genetic markers (BOLD markers and new specific designed marker) results using Geneious^®^ software with UPGMA method and the bootstrap values from 1000 replicates. The bar represents 0.006 substitutions per site

Compiling all the results from these three markers led to a new phylogenetic tree (Figure 4) that allowed differentiation of 6 lavender species:

*Lavandula pinnata*, *Lavandula stoechas* cv. “Pedunculata,” *Perovskia atriplicifolia*, *Lavandula canariensis*, *Lavandula latifolia* and *Lavandula angustifolia* cv. “Blue.”

The tree also showed two groups two samples groups with identical sequences for the three markers used:

The first group F included *Lavandula angustifolia* cv. “White” and the two lavandins (*Lavandula x intermedia* cv. "Abrial" and *Lavandula x intermedia* cv. "Grosso").

The second cluster, G, was composed of *Lavandula allardi* and *Lavandula dentata*.

## DISCUSSION

The aim of this study was the development of new method to authenticate lavender species in order to solve adulteration issues in lavender use. To do so, we combined chemical analysis (UHPLC) and genetic analysis (barcoding) that allowed to differentiate 6 lavender species.

### Chemical analysis

Chemical analysis enables us to identify 6 clusters and give first elements to differentiate several species of the genus *Lavandula*. Cluster 1 is characterized by the presence of compounds 8 and 14 as an important part in the discrimination of these group species. However, these compounds being volatile, they may be absent or irrelevant in older samples. For instance, it is well known that the quantity of coumaric acid (compound n° 8) can fluctuate according to the stage of development and time [42]. This compound is also involved in stress resistance mechanisms in plants, which can result in significant concentration fluctuations [43]. In addition, coumaric acid and ferulic acid hexoside may also take their origin from biotic stress response [44].

In this cluster, *Lavandula angustifolia* cv. “White” and *Lavandula x intermedia* cv. “Abrial” could also be differentiated. Indeed, the ratio of rosmarinic acid is close to the background noise or absent in these samples. In order to confirm this molecule as a marker of differentiation, a study of several development stages of each species would be necessary to conclude [45].

Rosmarinic acid is a member of the depsides family. Depsides produce phenolic acid which is furthermore studied by many research centres because of its proven antioxidant properties, immunostimulant, anti‐tumour, anti‐inflammatory and anti‐aggregation properties [46, 47, 48]. In this study, a majority of depsides is observed in *Lavandula stoechas* cv. “Pedunculata,” *canariensis* and *Perovskia atriplicifolia*. The presence of these molecules constitutes criterion 5 and allows us the differentiation of these species. Their presence is confirmed by recent literature, *Lamiaceae* family seems to be a rich source of plant species containing large quantities of depsides [49, 50].

Concerning criterion 3 identification, the presence of cinnamic acid‐derived molecules could be a good identification marker. Indeed, cinnamic acid derivatives are already known as plant authentication markers [51]. To confirm it, these markers will require quantification and analysis of multiple samples [52].

Compounds from different families may be present and may be related to contamination, adulteration or significant species differences. This is the case of the species *Perovskia atriplicifolia*, also called "Russian sage," which is closer to the species of *Salvia* sp. than other species of *Lavandula* sp. The presence of diterpenes is significantly different and excludes this sample from the panel. The *Lavandula stoechas* species also contains other compounds of this family (flavanone and terpene derivatives: compounds 48 and 69).

This chemical method allows us to identify molecules of interest in different lavender species. The presence or absence of some of them gives the keys to group the analysed samples in different clusters. In our study, coumaric acid hexoside, ferulic acid hexoside and rosmarinic acid are very interesting compounds and can be used as first elements to differentiate several species of the genus *Lavandula* and also to give clues for the identification of related species, especially those present in groups 1 and 2. However, phytochemical similarities between several samples and uncertainties related to natural variability (specific growth stages and conditions) did not allow a sure authentication of these species. These distinctions in the composition of secondary metabolites could be due to genetic modifications linked to the adaptation of these plant species to their environment. To confirm these results, further chemical analyses are necessary. In our study, we used genetic analysis to confirm and complete these results.

### Genetic analysis

In this study, we have shown that barcoding tool allows to identify 5 species of lavender and to discriminate with certainty two fine lavender at variety taxonomy level. *Lavandula allardi* and *dentata* species are in the same group. *Lavandula allardi* is the result of a genetic‐cross between *Lavandula angustifolia* and *Lavandula dentata* species which explains this result. This result is confirmed by [7]. It shows that the barcoding technique allows to eliminate the variation due to the natural variability or the stage of maturity of the plant. However, in our study we go further, differentiating two fine lavender (*Lavandula angustifolia*) varieties, blue and white from each other. Results also show a group including *L*. *angustifolia* cv. “White” variety and both lavandin that is the result of genetic‐cross with fine lavender [53]. These results are also confirmed by [54]. This result shows that the barcoding technique can in some cases allow us identification at the level of variety taxonomy. For further variety identification, microsatellite and snp markers will be needed. Our results allow us to go further with a set of three markers to analyse other lavender species. We used two BOLD DNA barcode primers for flowering plants, *RbcL* and *trnH*‐*psbA*, and we built specific *ITS* primer for this study [55, 56]. The ITS marker was designed specifically for this study and works specifically on those species of the genus *Lavandula* but it can also work on other botanical genera. These 3 markers distinguish *Lavandula pinnata*, *Lavandula stoechas* cv. “*Pedunculata*,” *Lavandula canariensis* and the outgroup *Perovskia atriplicifolia*. These data provide new information on the distinction of genus *Lavandula* species. We show that these three markers (*RbcL*, *trnH*‐*psbA* and ITS) are important to differentiate the species of the genus *lavandula*. So it is important to use several genetic markers to obtain an accurate identification of the species taxonomic rank. Indeed, it will be necessary to identify another genetic marker to differentiate species in groups F and G. The species included in these groups, are very close from genetic, chemical and morphological points of view.

The creation of specific barcoding markers or microsatellite or snp genetic markers could give the possibility to assign at the species level as confirmed by several scientific reports [57, 58]. However, morphological differences between species could result from post‐transcriptional modifications [59, 60]. In this case, a simple transcriptional analysis or an additional chemical analysis to quantify the presence of specific metabolites could differentiate them.

### Association of chemical analysis and genetic analysis

This study provides initial insights into the benefits of compiling genetic and chemical analyses in plant species authentication domain, as currently only chemical analyses are mandatory [3]. However, we know that there are limitations when only chemical authentication analyses are used, for specific raw materials or commercial products. Indeed, the results depend on the stage of development of the plant and the organ analysed [61, 62]. In addition, some molecules can be degraded in the time and cause different analysis results.

It is therefore sometimes difficult to obtain reproducible results between samples over time. On the other hand, the genetic approach does not depend on the stage of development of the plant or on the harvest conditions. Our study shows that genetic analysis allows us to make specific taxonomic identifications down to the variety level.

However, genetic analysis has some limitations on the identification of species or varieties. Indeed, some varieties or closely related species have adapted to particular environments (post‐transcriptional or translational modification) [59, 60]. This is the case of marine species that have adapted their metabolism to extreme saline conditions [63]. In this case, chemical analysis can provide additional information by identifying specific metabolites [64]. In order to ensure reliable results and to avoid misidentifications, it is important to combine genetic and chemical analyses [65, 66, 67]. Our study shows that the combination of both methods is a robust tool that will be important to develop in the future for taxonomic identification. However, it is important to note that in some cases only a genetic analysis is necessary as it is powerful and feasible with small amounts of material and gives reproducible results over time [68, 69]. We also show that genetic analysis allows us to obtain a precise taxonomic identification, up to the level of the variety. Therefore with these analytical methods, it is possible to control, maintain and improve the security of natural supply resources.

## CONCLUSIONS

Currently, plant authentication is of major importance to guarantee their origin and therefore their quality, traceability and transparency. We have set up an analytical system to discriminate between Lavandula species, which shows the role of genetic analysis. These analyses will address the challenges of authentication and traceability and ensure accurate and scientific confirmation of plant identity in materials from multiple sources.

## AUTHOR CONTRIBUTIONS

Nicole GIRAUD contributed to conceptualization; DNA Gensee Company (Florian PHILIPPE, Nelly DUBRULLE and Benjamin MARTEAUX) Methodology, Nicole GIRAUD and Florian PHILIPPE contributed to experimental and analysis part; and Nicole GIRAUD and Florian PHILIPPE contributed to writing—original draft. This work is the result of a cooperation model between private companies in the Cosmetics Industry who share the same committed and responsible approach to "act well together" in the service of higher stakes according to the United Nation's 17 SDG objectives.

## Supporting information

Data S1
